# Comparison between Ringer's lactate and balanced salt solution on postoperative outcomes after phacoemulsfication: A randomized clinical trial

**DOI:** 10.4103/0301-4738.49392

**Published:** 2009

**Authors:** Viraj Vasavada, Vaishali Vasavada, Nirmit V Dixit, Shetal M Raj, Abhay R Vasavada

**Affiliations:** Iladevi Cataract and IOL Research Centre, Raghudeep Eye Clinic, Gurukul Road, Memnagar, Ahmedabad, India

**Keywords:** Endothelium status, inflammation, irrigation fluid

## Abstract

**Aim:**

To compare the effects of balanced salt solution (BSS) and Ringer's lactate (RL) on corneal thickness, endothelial morphology, and postoperative anterior chamber inflammation in eyes undergoing phacoemulsification.

**Setting:**

Iladevi cataract and IOL research center, Ahmedabad, India.

**Materials and Methods:**

This prospective randomized study comprised 90 consecutive patients with age-related cataract who were randomly assigned to either Group 1 (n = 45) with BSS or Group 2 (n = 45) with RL. Observations made included measurement of central corneal thickness (CCT), presence of anterior chamber flare and cells, endothelial cell loss, and change in coefficient of variation (CV). Data was analyzed using Mann Whitney test and test of proportion.

**Results:**

Mean increase in CCT on postoperative Day 1 was 58 µm and 97 µm in Groups 1 and 2 respectively (*P* = 0.01). Increase in CCT at one month was 10µm and 11µm in Groups 1 and 2 respectively (*P* = 0.99); increase in CCT at three months was 3 µm and 6 µm in Groups 1 and 2 respectively (*P* = 0.86). Number of eyes with flare grades in a range of 0 to 3 was statistically higher in Group 2 on postoperative Day 1 (*P* = 0.004, 0.016, <0.001, 0.047 for Grade 0, 1, 2 and 3 respectively). Number of eyes with cells of Grade 3 on first postoperative day was significantly higher in Group 2 as compared to Group 1 (*P* = 0.004). Three months postoperatively, endothelial cell loss was 5.5% and 7.8% in Groups 1 and 2 (*P* = 0.21) and change in CV was 3 and 5.4 in Groups 1 and 2 (*P* = 0.20) respectively.

**Conclusion:**

BSS offers a significant advantage over RL in terms of increase in corneal thickness and postoperative inflammation on the first postoperative day in patients undergoing phacoemulsification.

During cataract surgery, replacing the aqueous fluid with an irrigating solution can affect the survival and functioning of the endothelial cells. The safety of an irrigating solution, especially when used for extended periods, is critically dependent on its composition.[1,2] Balanced salt solution Plus (BSS Plus) has an ionic composition similar to that of the aqueous fluid along with additional constituents such as oxidized glutathione. The results of several experimental corneal perfusion and clinical studies suggest that a more physiological solution (BSS Plus) may prove to be a better infusion solution for intraocular surgery than others presently being used such as (balanced salt solution (BSS) and Ringer's lactate (RL).[3–8]

The high cost of BSS Plus limits its widespread acceptability and usage. On the other hand, despite the fact that RL lacks several essential constituents necessary for endothelial functioning and protection, it remains the most widely used irrigating fluid in our part of the world due to its low cost. BSS has a chemical composition superior to RL; it contains magnesium (essential for the Mg-ATPase endothelial pump) and an acetate citrate buffer system in addition to potassium, calcium, and lactate. BSS is only slightly hypotonic to the aqueous fluid and has an alkaline pH. It can be considered as a cost-effective alternative to BSS Plus. Edelhauser *et al.*[4] in an experimental setting perfused corneas with RL and BSS. They found that corneas perfused with lactated Ringer solution swell at a faster rate and the endothelial cells show degenerative changes earlier as compared to corneas perfused with BSS.

However, there have not been many studies comparing the effect of BSS and RL in a clinical scenario. Therefore, in this study, we attempted to compare the effect of BSS versus RL on the corneal thickness, morphology, and postoperative anterior chamber inflammation following phacoemulsification.

## Materials and Methods

This prospective, randomized examiner and patient masked study comprised 90 eyes of 90 patients with uncomplicated senile cataract, aged between 55 to 70 years, scheduled for phacoemulsification. Informed consent was obtained from all patients before they were enrolled in the study. The study followed the tenets of Helsinski. Patients with coexisting corneal pathologies, an endothelial cell count of <1500 cells/mm^3^, glaucoma, diabetes mellitus, uveitis, trauma, or prior intraocular surgery were excluded from the study. Other exclusion criteria were patients on systemic or topical steroids. All types of cataract and grades of nuclear sclerosis ranging on a scale of 1 to 4 as per Emery's classification were included in the study.[9]

The preoperative investigation included a slit-lamp examination and fundus evaluation. Specular microscopy was performed with a non-contact specular microscope (Topcon, Tokyo, Japan) to analyze the central corneal endothelium. One hundred contiguous cells were analysed.[10] The endothelial cell density (ECD) and coefficient of variation (CV) were measured in all cases. CV was calculated by dividing the standard deviation of the cell areas measured in each specular micrograph by the mean cell area. This index provided a measurement of the cell size variability (polymegathism) which is independent of cell area and density. Central corneal thickness (CCT) was assessed by ultrasonic pachymetry (Ocuscan, Alcon, Texas, USA) performed by a single observer. The subject was asked to look at a fixation target placed at a certain distance while the ultrasound probe was placed perpendicular to the central cornea without applying any undue pressure. An average of three readings was considered.[11] Prior to surgery, the patients were randomized into two groups using computer-generated random numbers. Group 1 received BSS and Group 2 received RL as an irrigating solution [Table 1] during surgery. The fluids were stored in a room and brought inside the operation theater immediately prior to the surgery to maintain a temperature of 23 degrees celcius during surgery.

**Table 1:** A comparison of the preoperative corneal profile and intraoperative performance between the two groups

		BSS	Ringer's lactate	*P* value T-test
Preoperative central corneal thickness (CCT- µm)	Mean	533	523	0.09
	Median	536	522	
	S.D.	27	30	
Preoperative endothelial cell density(ECD)	Mean	2438	2532	0.09
	Median	2508	2479	
	S.D.	311	221	
Preoperative coefficient of variation (CV)	Mean	44	44	1.00
	Median	43	43	
	S.D.	8	8	
Surgical clock time (min) (in minutes)	Mean	5.08	5.2	0.87
	Median	5	5	
	S.D.	2.18	2.76	
Fluid used (ml) (in ml)	Mean	110	118	0.40
	Median	111	100	
	S.D.	40	55	
Effective phaco time (EPT)	Mean	54	53	0.86
	Median	49	51	
	S.D.	23	36	

BSS-Balanced salt solution

A single surgeon (ARV) performed all surgeries under topical anesthesia using a standardized surgical technique. Two limbal paracentesis incisions of 1mm were made in the clear cornea 180 degrees apart using an angled dual beveled 1-mm knife (Alcon Laboratories, Fort Worth, USA). Viscoat^®^ (Alcon Laboratories, Fort Worth, USA) was injected into the anterior chamber to coat the corneal endothelium followed by the injection of Provisc (Alcon laboratories, USA) to maintain the depth of the anterior chamber.[12] A 2.2-mm single plane temporal clear corneal incision was made. A bent 26G cystotome was used to initiate a small nick in the anterior capsule and capsulorrhexis was completed using Uttrata forceps. Thorough multiquadrant hydrodissection was performed. Microcoaxial phacoemulsification was performed on the Infiniti Vision System (Alcon Laboratories, Fort Worth, USA) using step-by-step, chop *in situ*, lateral separation[13] and step-down[14] techniques.

The parameters used during different stages of surgery were a power of 30–50%, microburst with burst width of 5–30 milliseconds, a vacuum of 250–650 mm Hg, and an aspiration flow rate of 25–30 cc/min. The bottle height was raised to a maximum of 110 cm from a minimum of 90 cm during fragment removal. Care was taken to perform phacoemulsification at the posterior plane. Constant anterior chamber depth was maintained with injection Viscoat^®^ before removing any instrument from the eye. Bimanual irrigation/aspiration was performed for cortex removal. An Acrysof Natural IQ intraocular lens (IOL) (SN60WF, 6 mm optic, 13 mm overall diameter) was implanted in the bag. The residual viscoelastic was removed with bimanual irrigation/aspiration. The main incision as well as the paracentesis was hydrated.[15] The postoperative regime was also standardized in both groups.

The following intraoperative observations were made: amount of fluid used from commencement of sculpting to the end of viscoelastic removal after IOL implantation, surgical clock time from commencement of sculpting to the end of epinucleus removal, effective phaco time (average ultrasound (u/s) power × average u/s time/100), occurrence of excessive iris manipulation, Descemet's detachment, incisional burns, and posterior capsule rupture.

Postoperatively, the patient was examined on Day 1, and at one and three months. At each follow-up visit, corneal clarity was noted as the presence or absence of Descemet's folds and intrastromal or epithelial edema. Anterior chamber flare and cells were graded as per Hogan's criteria.[16] The CCT was measured by ultrasound pachymetry at postoperative Day 1 and at one and three months postoperatively. At three months follow-up, specular microscopy was performed. These examinations were done by the same observer who performed the tests preoperatively and was masked to the allocation.

The main outcome measures were an increase in CCT from the baseline preoperative value, percentage loss of ECD, change in the CV from preoperative values, and change in the anterior chamber flare and cells between the groups.

The increase in CCT in microns was calculated by noting the difference between CCT on the first postoperative day and the preoperative CCT. Similarly, change in CCT was also calculated at the one and three months follow-up visits. Percentage decrease in endothelial cell density (ECD) was calculated as follows:

$$\frac{\text{ECD at 3 months follow-up}-\text{preoperative ECD}\times 100}{\text{Preoperative ECD}}$$

The change in the CV was calculated as the difference between CV at the three months follow-up and the preoperative CV.

Data were entered in an Excel file and computed using a statistical software package (SPSS 3.1, SPSS Inc, Chicago). Statistical analysis was done using the non-parametric Mann-Whitney test, as the data were not normally distributed and 95% confidence intervals were calculated for differences in mean results. A *P* value of <0.05 was considered statistically significant.

## Results

Of the 90 patients enrolled in the study, 83 patients completed the last postoperative follow-up. Two patients were transferred to a distant destination, one suffered an acute ischemic heart disease and expired. One patient refused to continue with his participation in the study as a macular hole was detected during a follow-up visit. Two patients were recuperating from illness and therefore their results were excluded from the analysis. The groups were comparable in terms of gender, age, and distribution of the type and density of cataract. The mean age of the patients was 58 ± 13.3 years in Group 1 and 56.5 ± 15.7 years in Group 2. The mean follow-up was 3.1 ± 0.2 months and 2.9 ± 0.2 months in Group 1 and 2 respectively.

The preoperative CCT in both the groups was comparable (533 + 27 in BSS versus 523 + 31 um in RL). The surgical clock time and amount of fluid used in both the groups was also comparable [Table 1]. In both the groups, the CCT increased from the preoperative baseline on the first postoperative day. This increase was significantly higher in Group 2 as compared with Group 1 [Table 2]. There was no significant difference in CCT between the groups at the one month or three month follow-up.

**Table 2:** A comparison of the postoperative outcome in both the groups

		BSS	Ringer lactate	*P* value of Mann-whitney
Increase in central corneal thickness (CCT) at first postoperative day (in µm)	Mean	58	97	0.01
	Median	53	79	
	S.D.	34	67	
Increase in central corneal thickness (CCT) at one month (in µm)	Mean	10	11	0.99
	Median	8.6	8.2	
	S.D.	23	28	
Increase in central corneal thickness (CCT) at three months (in µm)	Mean	3	6	0.86
	Median	2	2	
	S.D.	11	19	
Endothelial cell loss at three months (%)	Mean	5.5	7.8	0.21
	Median	7.1	8	
	S.D.	5.5	10.3	
Change in coefficient of variation (CV) at three months	Mean	3	5.4	0.20
	Median	3	5	
	S.D.	7	9	

The mean endothelial cell (ECD) loss at three months was 5% in Group 1 and 8% in Group 2. The mean change in CV from preoperative values was 3 in Group 1 and 5 in Group 2. The ECD loss and change in CV at three months were statistically insignificant.

On postoperative Day 1, none of the eyes in either group had flare of Grade 4 severity. A higher percentage of individuals in the BSS group had statistically significant lower levels of flare [Table 3]. The percentage of individuals with cells of Grade 3 severity was significantly higher in the group subjected to RL. In subsequent examinations, flare and cells were not evident in any eye of either group.

**Table 3:** Percentage of individuals with flare and cells on the first postoperative day in both the groups

Grade	Flare			Cells		
		
	BSS	RL	*P* value of test of proportion	BSS	RL	*P* value of test of proportion
0	24	4	0.004*	5	0	0.11
1	53	29	0.016*	47	36	0.27
2	18	50	0.000*	37	25	0.20
3	5	18	0.047*	11	36	0.004*
4	0	0	-	0	4	0.16

*P* < 0.05 was considered as statistically significant, Flare was graded as: 0 = 0, 1 = faint – just detectable, 2 = moderate – iris details clear, 3 = marked – iris details hazy, 4 = intense – with severe fibrinous exudates, Cells were graded as: 0 = no cells, 1 = 5–10 cells, 2 = 11–20 cells, 3 = 21–50 cells, 4 = >50 cells

## Discussion

A majority of clinical studies have compared BSS Plus to BSS.[2,6,17,18] This study was confined to a comparison between RL and BSS because of the widespread use and cost-effectiveness of RL in comparison to BSS Plus. Moreover, comparing RL and BSS would be clinically more relevant in the context of minimally traumatic modern cataract surgeries. We chose not to include BSS Plus in the comparison because sufficient evidence already exists about its ability to induce minimal corneal alterations both in experimental[1,2,19] and clinical trials[18,20] and also because of its selective use owing to its high cost.

Ringer lactate contains potassium, calcium, and lactate ions which maintain corneal endothelial cells for long periods. Calcium is essential for protecting the endothelial cell functions.[21] However, RL is hypotonic and slightly acidic (osmolality 280mmol, pH 6.0) as compared to BSS (osmolality 302 mmol, pH 7.4) and aqueous (osmolality 302 mmol, pH 7.4). It lacks a buffer system and energy source for the endothelium. In addition to potassium, calcium, and lactate, BSS contains magnesium (essential for the Mg-ATPase endothelial pump) and an acetate citrate buffer system. It is only slightly hypotonic to the aqueous fluid and has an alkaline pH. Previous studies have shown that BSS is superior to RL, though not as ideal as BSS Plus.[4]

Corneal thickness increases when the pump and barrier functions of the endothelium are compromised. Measuring CCT helps gauge the extent of the surgically induced endothelial trauma. There was an acute, significant increase in the CCT on postoperative Day 1 in both the groups. BSS induced significantly lower corneal swelling than RL on the first postoperative day. The increase in CCT observed on the first postoperative day and its reversal to values close to the preoperative baseline at three months concurs with the observations of Kiss *et al*.[6] Standardization of the surgical technique by a single experienced surgeon eliminated mechanically induced trauma as a cause for endothelial damage. The similarity in the distribution of type and grade of nuclear sclerosis eliminated energy dissipation as a cause for increased corneal thickness. Since the duration of the surgical time and amount of fluid used was comparable in both the groups, differences in the constitution appear to be responsible for higher short-term damage to the corneal endothelium in the RL group. This is reflected by the increased corneal swelling in the immediate postoperative period in the group subjected to RL.[4] This may, in turn, delay visual rehabilitation. In addition, the increased corneal swelling with RL may be even more significant in eyes with compromised corneas.

The average decrease in postoperative endothelial cell density was comparable to values observed in previous studies.[22,23] There was no difference in terms of cell density between the two groups at three months postoperatively. The changes in the morphology of the endothelial cells are sensitive indicators of loss of endothelial function.[24] The CV of cell areas (polymegathism) was determined by measuring 100 cells in each group. It was observed that both the groups had similar compositions of small and large cells. The Viscoat^®^ used in every case may have provided substantial barrier protection that resulted in the lack of significant difference in endothelial cell counts between the two groups.[25]

A majority of studies have evaluated the impact of irrigating fluids on the corneal endothelial function. However, very few studies have evaluated postoperative inflammation.[17,26] The use of cooled irrigating fluids has been recommended as a method of reducing postoperative inflammation.[26] However, such cooled fluids have only a short-term impact. The presence of a low grade of flare and cells on the first postoperative day in a majority of our patients in the BSS group supports our hypothesis that cooling the irrigating fluid is not necessary to reduce inflammation.[27] Since the surgeries were done by a single surgeon experienced in phacoemulsification, the difference in the inflammatory response can be solely attributed to the composition of the fluid. Our results concur with the favorable response on inflammation observed with BSS. We believe that a statistically significant larger number of patients with flare and cells of Grades 2 and 3 in Group 2 on the first postoperative day may be due to the slightly acidic fluctuating pH and lack of buffer system in RL which could affect the blood aqueous barrier stability. On the other hand, BSS has an acetate citrate buffer system and the pH, though slightly alkaline, is stable.

This is one of the few studies evaluating the impact of irrigating fluids for a postoperative duration of three months.[6] The present study has shown the clinical advantages of BSS in the immediate postoperative period and its use seems justified in eyes undergoing cataract surgery. Along with appropriate technique and technology, the clinical benefit offered by BSS on the first postoperative day helps the patient achieve clear cornea on Day 1 with immediate visual rehabilitation. We believe the impact of using RL in eyes with low endothelial reserve can be magnified. Therefore, BSS should atleast be considered in lieu of RL in selective eyes with a lower endothelial cell reservoir, anticipated prolonged surgical duration, and those requiring excessive surgical manipulation.

In conclusion, the results of this prospective randomized masked trial demonstrated that eyes receiving BSS had significantly lesser corneal thickness and inflammation on the first postoperative day as compared to eyes that received RL, however, there was no significant difference at one and three months postoperatively.

## References

[1] Edelhauser HF, Gonnering R, Van Horn DL (1978). Intraocular irrigating solutions. A comparative study of BSS Plus and lactated Ringer's solution. Arch Ophthalmol.

[2] Glasser DB, Matsuda M, Ellis JG, Edelhauser HF (1985). Effects of intraocular irrigating solutions on the corneal endothelium after *in vivo* anterior chamber irrigation. Am J Ophthalmol.

[3] Yagoubi MI, Armitage WJ, Diamond J, Easty DL (1994). Effects of irrigation solutions on corneal endothelial function. Br J Ophthalmol.

[4] Edelhauser HF, Van Horn DL, Hyndiuk RA, Schultz RO (1975). Intraocular irrigating solutions: Their effect on the corneal endothelium. Arch Ophthalmol.

[5] Puckett TR, Peele KA, Howard RS, Kramer KK (1995). Intraocular irrigating solutions: A randomized clinical trial of balanced salt solution plus and dextrose bicarbonate lactated Ringer's solution. Ophthalmology.

[6] Kiss B, Findl O, Menapace R, Petternel V, Wirtitsch M, Lorang T (2003). Corneal endothelial cell protection with a dispersive viscoelastic material and an irrigating solution during phacoemulsification: Low-cost versus expensive combination. J Cataract Refract Surg.

[7] Kramer KK, Thomassen T, Evaul J (1991). Intraocular irrigating solutions: A clinical study of BSS plus and dextrose bicarbonate lactated Ringer's solution. Ann Ophthalmol.

[8] Matsuda M, Tano Y, Edelhauser HF (1984). Comparison of intraocular irrigating solutions used for pars plana vitrectomy and prevention of endothelial cell loss. Jpn J Ophthalmol.

[9] Emery JM, Little JH (1979). Phacoemulsification and as per of cataracts, surgical technique, complications and results.

[10] Hurst LW, Ferris FL, Stark WJ, Fishman JA (1980). Clinical specular microscopy (endothelial). Invest Ophthalmol Vis Sci.

[11] Tam ES, Routman DS (2003). Comparison of central corneal thickness measurements by specular microscopy, ultrasound pachymetry and ultrasound biomicroscopy. J Cataract Refract Surg.

[12] Arshinoff SA (1999). Dispersive cohesive viscoelastic soft shell technique. J Cataract Refract Surg.

[13] Vasavada AR, Singh R (1998). Step by step chop in situ and separation of very dense cataracts. J Cataract Refract Surg.

[14] Vasavada AR, Raj S (2003). Step down technique. J Cataract Refract Surg.

[15] Vasavada AR, Praveen MR, Pandita D, Gajjar DU, Vasavada VA, Vasavada VA (2007). Effect of stromal hydration of clear corneal incisions: Quantifying ingress of trypan blue into the anterior chamber after phacoemulsification. J Cataract Refract Surg.

[16] Kanski JJ (1994). Uveitis. Clinical ophthalmology: A systematic approach, 3 Rev.ed. Clinical features of uveitis.

[17] Stoldt G, Struck HG (1997). Different ophthalmologic surgery irrigation solutions: Studies of postoperative inflammation. Ophthalmologe.

[18] Joussen AM, Barth U, Cubuk H, Koch H (2000). Effect of irrigating solution and irrigation temperature on the cornea and pupil during phacoemulsification. J Cataract Refract Surg.

[19] Nemet AY, Assia EI, Meyerstein D, Meyerstein N, Gedanken A, Topaz M (2007). Protective effect of free-radical scavengers on corneal endothelial damage in phacoemulsification. J Cataract Refract Surg.

[20] Matsuda M, Kinoshita S, Ohashi Y, Shimomura Y, Ohguro N, Okamoto H (1991). Comparison of the effects of intraocular irrigating solutions on the corneal endothelium in intraocular lens implantation. Br J Ophthalmol.

[21] Koye GI, Mishima S, Cole JD, Kaye NW (1968). Studies on the cornea: VII Effects of perfusion with Ca-free medium on the corneal endothelium. Invest Ophthalmol.

[22] Dholakia SA, Vasavada AR, Singh R (2005). Prospective evaluation of phacoemulsification in adults younger than 50 years. J Cataract Refract Surg.

[23] Dholakia SA, Vasavada AR (2004). Intraoperative performance and long term outcome of phacoemulsification in age-related cataract. Indian J Ophthalmol.

[24] McCarey BE, Edelhauser HF, Van Horn DL (1973). Functional and structural changes in the corneal edothelium during *in vitro* perfusion. Invest Ophthalmol.

[25] Koch DD, Liu JF, Glasser DB, Merin LM, Haft E (1993). A comparison of corneal endothelial changes after use of Healon or Viscoat during phacoemulsification. Am J Ophthalmol.

[26] Findl O, Amon M, Kruger A, Petternel V, Schauersberger J (1999). Effect of cooled intraocular irrigating solution on the blood-aqueous barrier after cataract surgery. J Cataract Refract Surg.

[27] Praveen MR, Vasavada AR, Shah R, Vasavada VA (2008). Effect of room temperature and cooled intraocular irrigating solution on the cornea and anterior segment inflammation after phacoemulsification: A randomized clinical trial. Eye.

